# Prostate‐specific antigen nadir within 12 months as an early surrogate marker of biochemical failure and distant metastasis after low‐dose‐rate brachytherapy or external beam radiotherapy for localized prostate cancer

**DOI:** 10.1002/cam4.1443

**Published:** 2018-03-25

**Authors:** Shuichi Nishimura, Toshio Ohashi, Tetsuo Momma, Masanori Sakayori, Takahisa Eriguchi, Tomoki Tanaka, Shoji Yamashita, Takeo Kosaka, Mototsugu Oya, Naoyuki Shigematsu

**Affiliations:** ^1^ Department of Radiology Keio University School of Medicine Tokyo Japan; ^2^ Department of Urology National Hospital Organization Saitama Hospital Saitama Japan; ^3^ Department of Radiology National Hospital Organization Saitama Hospital Saitama Japan; ^4^ Department of Urology Keio University School of Medicine Tokyo Japan

**Keywords:** Biochemical failure, brachytherapy, distant metastasis, prostate cancer, prostate‐specific antigen nadir, radiotherapy

## Abstract

Prostate‐specific antigen nadir (nPSA) after radiotherapy for localized prostate cancer has been investigated as a predictor. However, nPSA usually requires several years, limiting its clinical utility. We investigated the significance of nPSA within 12 months (nPSA12) after low‐dose‐rate prostate brachytherapy (LDR‐PB) or external beam radiotherapy (EBRT) on treatment outcomes. Between 2006 and 2014, 663 patients with prostate cancer were treated with LDR‐PB or EBRT at two institutions. Four hundred and seventy‐four men received LDR‐PB and 189 men received EBRT, without androgen deprivation therapy. The Kaplan–Meier method was used for biochemical failure (BF)‐free survival (BFFS) and distant metastasis (DM)‐free survival (DMFS) analyses, and multivariable Cox regression analysis was performed. The median follow‐up was 61.3 months. The median nPSA12 in the LDR‐PB and EBRT cohorts was 0.7 and 1.0 ng/mL, respectively. The 7‐year BFFS and DMFS rates in LDR‐PB patients with nPSA12 ≤ 0.7 ng/mL were 99.1% and 99.5%, respectively; when nPSA12 was >0.7 ng/mL, they were 90.2% and 94.8%, respectively. In EBRT patients with nPSA12 ≤ 1.0 ng/mL, BFFS and DMFS rates were 85.4% and 98.5%, respectively; when nPSA12 was >1.0 ng/mL, they were 67.1% and 87.2%, respectively. nPSA12 was an independent predictor of BF and DM in both cohorts (LDR‐PB,* P* = 0.004 and 0.020, respectively; EBRT,* P* = 0.005 and 0.041, respectively). The nPSA12 after LDR‐PB or EBRT is significantly associated with treatment outcomes of prostate cancer. Higher nPSA12 may identify patients at high risk of relapse who might benefit from salvage treatment.

## Introduction

Serum prostate‐specific antigen (PSA) is an important tool for prostate cancer screening and a marker for treatment response and disease recurrence. PSA nadir (nPSA) after definitive radiotherapy for prostate cancer has been investigated as a predictor of treatment outcomes that is independent of pretreatment clinical factors, including pretreatment PSA, Gleason score, T‐stage, and radiation dose 1, 2, 3, 4, 5, 6, 7. Some studies have also suggested that the time to nPSA after radiotherapy is associated with treatment outcomes 1, 7, 8. However, nPSA usually requires several years before determination, limiting its clinical utility as a predictive factor. Earlier markers of recurrence risk would be clinically useful. A few reports have shown that time‐limited measures of PSA are early significant predictors of biochemical failure (BF) and distant metastasis (DM) that are independent of other predictive clinical factors of treatment outcomes after definitive external beam radiotherapy (EBRT), while it remains to be investigated after low‐dose‐rate prostate brachytherapy (LDR‐PB) 9, 10, 11.

The purpose of this study was to determine the significance of nPSA within 12 months (nPSA12) in predicting BF and DM in patients treated with LDR‐PB or definitive EBRT for localized prostate cancer.

## Materials and Methods

### Patient population and treatment strategy

A total of 663 consecutive patients with localized prostate cancer (cT1‐T4, N0, M0) were included in this retrospective study. Patients were treated with LDR‐PB ± EBRT or definitive EBRT alone without androgen deprivation therapy (ADT) from June 2006 through October 2014 at either the Keio University School of Medicine or the National Hospital Organization Saitama Hospital, with a minimum follow‐up duration of 24 months. Patients were classified into risk groups according to National Comprehensive Cancer Network guidelines. LDR‐PB was initiated in January 2007. LDR‐PB alone was offered only to low‐risk patients (T1‐T2a, PSA <10 ng/mL, and Gleason score ≤6) and low‐tier intermediate‐risk patients (T2b‐c, PSA <10 ng/mL, and Gleason score of 3 + 4 with a biopsy‐positive core rate <33%). LDR‐PB + EBRT was administered primarily to intermediate‐ to high‐risk patients. Conventional EBRT and intensity‐modulated radiation therapy (IMRT) were administered to patients in all risk categories. IMRT was initiated in December 2007. Since then, the use of conventional EBRT has been largely replaced by IMRT. There were no treatment policy discrepancies between the two participating institutes. The institutional review board at each institution approved this study.

### Conventional EBRT

For three‐dimensional conformal EBRT (3D‐CRT), the clinical target volume (CTV) included either the entire prostate or the prostate and seminal vesicles, depending on the risk category. The planning target volume (PTV) was defined by adding a 1‐cm margin around the CTV, except posteriorly at the rectum interface, where a 6‐mm margin was used. A four‐field box technique was used, in which a multileaf collimator‐defined radiation beam aperture conformed to the PTV with the appropriate margins. All treatments were delivered using 6‐ to 10‐MV photons. The median dose was 70 Gy (range, 70–74 Gy) in 2‐Gy fractions at the isocenter.

### IMRT

The CTV for IMRT was the same as for EBRT. The PTV was defined by adding a 7‐mm margin around the prostate except posteriorly at the rectal interface, where a 6‐mm margin was used, and by adding a 5‐mm margin around the seminal vesicles. The rectum and bladder were delineated as solid organs. The rectum was contoured from 4 mm above the PTV to 4 mm below the PTV. A five‐ to seven‐field step‐and‐shoot IMRT plan was created using 10‐MV photons. The prescribed radiation dose represented the minimum dose to 95% of the PTV. The median doses were 78 Gy (range, 70–80 Gy). Dose constraints included a maximum dose to the PTV ≤107% of the prescribed dose, mean dose to the PTV ≥100% of the prescribed dose, the dose delivered to 95% of the PTV ≥100% of the prescribed dose, maximum dose to the rectum ≤107% of the prescribed dose, the dose delivered to 1% of the rectum ≤78 Gy, the dose delivered to 5% of the rectum ≤76 Gy, the dose delivered to 20% of the rectum ≤60 Gy, the dose delivered to 40% of the rectum ≤40 Gy, and the maximum dose to the bladder ≤107% of the prescribed dose.

### LDR‐PB ± EBRT

The techniques and dose constraints used for the LDR‐PB and LDR‐PB + EBRT groups have been previously described 12, 13, 14. The prescribed dose was 160 Gy for LDR‐PB alone and 110 Gy for LDR‐PB + EBRT. The PTV was defined as the prostate itself. Dose constraints included V100 > 95%, V150 < 50%, 110% < D90 < 130%, urethral volume receiving 150% of the prescribed dose <0.1 cc, and rectal volume receiving 100% of the prescribed dose <0.1 cc. Postimplant computed tomography (CT) dosimetry was performed 1 month after implantation, with doses converted to biologically effective dose (BED) from the postplan D90 using an *α*/*β* ratio of 2. In the LDR‐PB + EBRT group, approximately 4–8 weeks after seed implantation, supplemental EBRT was initiated using 3D‐CRT with 10‐MV photons to a median dose of 45 Gy in 1.8‐Gy fractions at the isocenter.

### Follow‐up and end points

The date of brachytherapy implantation or EBRT completion was considered day 0 for follow‐up duration calculations. Clinical follow‐up evaluations after treatment were performed at 3‐month intervals for 2 years and every 6 months thereafter. The nPSA12 was defined as the lowest PSA level within 12 months after LDR‐PB or EBRT completion. Therefore, nPSA12 was the lowest value among the 3‐month, 6‐month, 9‐month, and 12‐month PSA levels. BF was defined according to the Phoenix definition (nPSA + 2 ng/mL). DM was confirmed by subsequent bone scan, CT imaging, or both.

### Statistical analysis

The chi‐square test or unpaired t‐test was used to compare patient characteristics and clinical factors between low‐ and high‐nPSA12 groups. Estimates of BF‐free survival (BFFS) and DM‐free survival (DMFS) rates were calculated using the Kaplan–Meier method. To identify the effect of potential prognostic factors on treatment outcome, univariate analysis was performed using the log‐rank test. Variables with *P* < 0.25 were entered into multivariate analysis (MVA) using the Cox proportional hazards model. Pearson correlation coefficient was used to assess the relation between pretreatment PSA and nPSA12. Statistical analyses were performed using SPSS, version 23.0 (IBM, Chicago, IL). All tests were two‐sided, and statistical significance was set at the level of *P* < 0.05.

## Results

### Patient characteristics and cutoff point for nPSA12

Of the 663 patients, 474 received LDR‐PB ± EBRT (LDR‐PB cohort), and 189 received definitive EBRT alone (EBRT cohort). The patient characteristics are listed in Table 1, including pretreatment PSA, Gleason score, T‐stage, and total BED or prescribed dose. The median follow‐up duration for all patients was 61.3 months. The median nPSA12 in the LDR‐PB and EBRT cohorts was 0.7 and 1.0 ng/mL, respectively. There were statistically significant differences between the low‐ and high‐nPSA12 groups in the LDR‐PB cohort in median follow‐up, patient age, Gleason score, and supplemental EBRT. There were significant differences in pretreatment PSA in the EBRT cohort. The optimal cutoff for nPSA12 has not been previously determined. Using a high nPSA12 cutoff is inadvisable because the proportion of patients with unfavorable nPSA12 values becomes considerably small. Therefore, the median nPSA12 values were used in this study.

**Table 1:** Patient characteristics

	LDR‐PB (*n* = 474)				EBRT (*n* = 189)			
	All	nPSA12 ≤0.7 ng/mL	nPSA12 >0.7 ng/mL	*P*	All	nPSA12 ≤.0 ng/mL	nPSA12 >1.0 ng/mL	*P*
Median follow‐up (months)	61.2	59.6	62.0	0.004	61.8	60.4	64.4	0.523
Age (years)								
Median	70	71	68	<0.001	71	72	70	0.124
Range	51–82	55–82	51–80		41–86	41–86	54–83	
Pretreatment PSA (ng/mL)								
<10	387	198	189	0.126	118	68	50	0.007
10–20	83	42	41		57	20	37	
>20	4	0	4		14	5	9	
Gleason score								
≤6	301	138	163	0.019	51	19	32	0.117
7	152	91	61		111	60	51	
8–10	21	11	10		27	14	13	
T‐stage								
≤T2a	429	213	216	0.149	123	61	62	0.823
T2b–T2c	42	24	18		32	16	16	
≥T3a	3	3	0		34	16	18	
Supplemental EBRT								
Yes	168	111	57	0.003				
No	286	129	157					
Total BED (Gy_2_)								
Median	214	215	213	0.229				
Range	152–259	152–259	162–257					
Prescribed dose (Gy)								
Median					78	78	76	0.585
Range					70–80	70–80	70–80	

LDR‐PB, low‐dose‐rate prostate brachytherapy; EBRT, external beam radiotherapy; PSA, prostate‐specific antigen; nPSA12, prostate‐specific antigen nadir within 12 months; BED, biologically effective dose.

### Low nPSA12 is associated with favorable clinical outcomes

**Figure 1:**
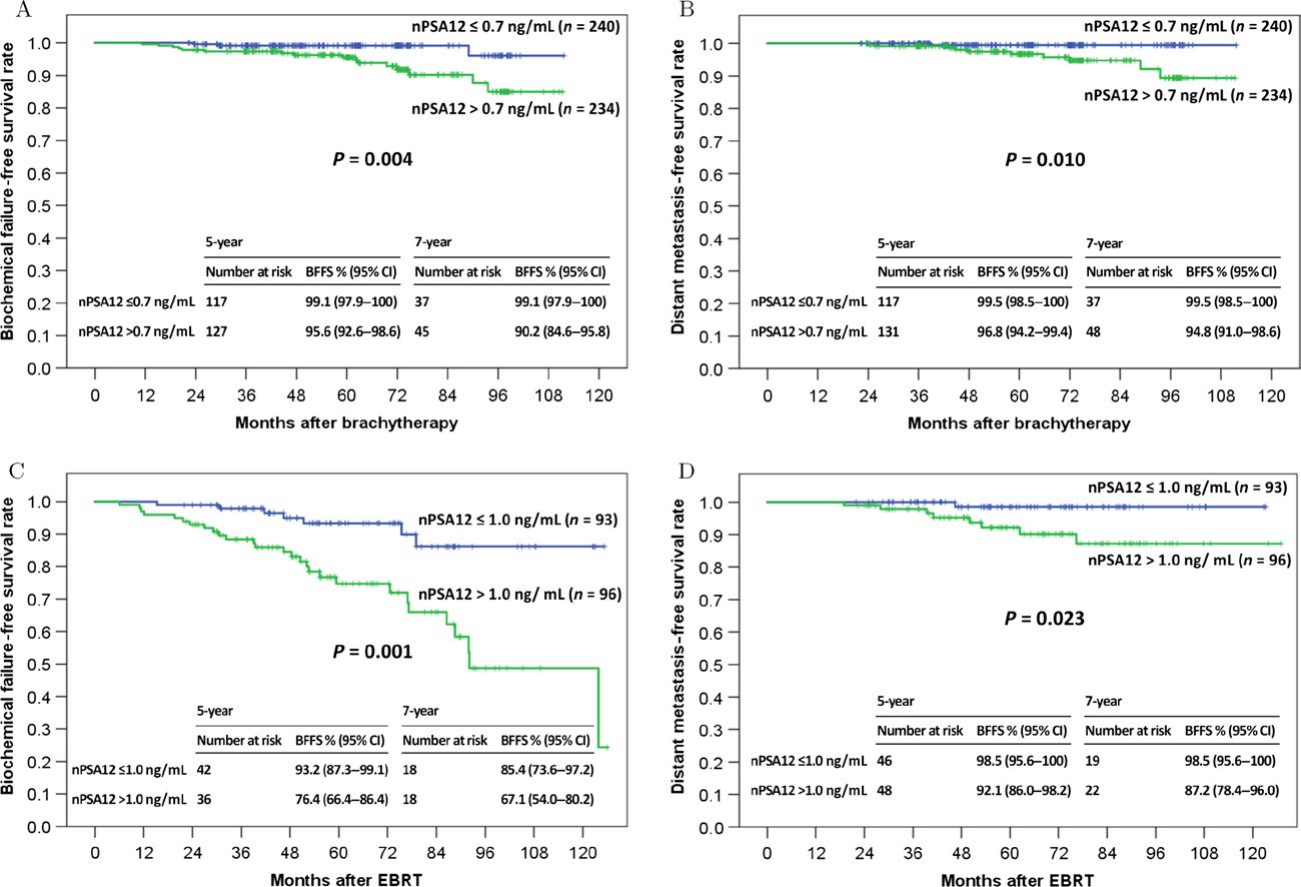
Kaplan–Meier survival curves. (A) Biochemical failure‐free survival (BFFS) and (B) distant metastasis‐free survival (DMFS) of patients who underwent low‐dose‐rate prostate brachytherapy according to the level of prostate‐specific antigen nadir within 12 months (nPSA12). (C) BFFS and (D) DMFS of patients who underwent external beam radiotherapy (EBRT) according to nPSA12. There were significant differences in these outcomes between patients with a low nPSA12 (≤median) and those with a high nPSA12 (>median). CI, confidence interval.

Patients with high nPSA12 had significantly inferior BFFS and DMFS compared to patients with low nPSA12 in both cohorts. The 7‐year BFFS rates for patients in the LDR‐PB cohort with nPSA12 ≤ 0.7 and >0.7 ng/mL were 99.1% and 90.2%, respectively (*P* = 0.004), and the 7‐year DMFS rates were 99.5% and 94.8%, respectively (*P* = 0.010) (Fig. 1A and B). The 7‐year BFFS rates for patients in the EBRT cohort with nPSA12 ≤ 1.0 and >1.0 ng/mL were 85.4% and 67.1%, respectively (*P* = 0.001), and the 7‐year DMFS rates were 98.5% and 87.2%, respectively (*P* = 0.023) (Fig. 1C and D). The median months to BF and DM in the LDR‐PB cohort were 47.4 and 44.9, respectively, and they were 46.4 and 30.5, respectively, in the EBRT cohort.

**Figure 2:**
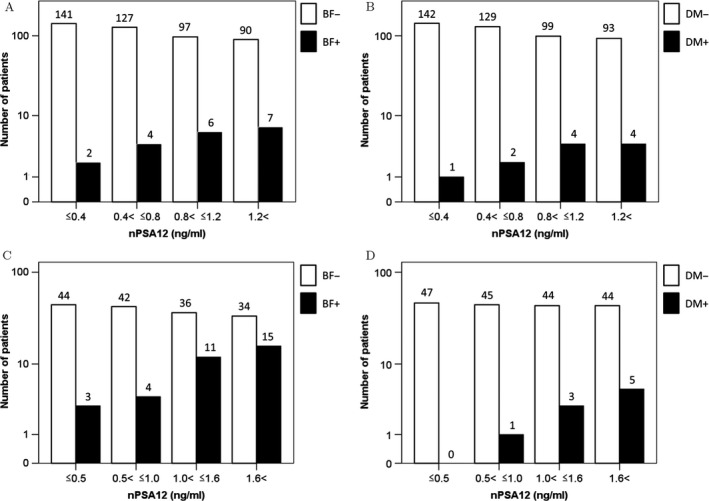
Distribution of prostate‐specific antigen nadir within 12 months (nPSA12) according to clinical control of (A) biochemical failure (BF) and (B) distant metastasis (DM) of patients who underwent low‐dose‐rate prostate brachytherapy. Distribution of nPSA12 according to clinical control of (C) BF and (D) DM of patients who underwent external beam radiotherapy. The rates of relapse increase with higher nPSA12 in each cohort.

Figure 2 demonstrates the distribution of patients with or without BF and DM according to the nPSA12 achieved in the two cohorts. The rates of BF and DM increased in the higher nPSA12 quartile groups in both cohorts.

### Clinical factors affecting BFFS and DMFS

MVA in the LDR‐PB cohort demonstrated that nPSA12 (≤0.7 vs. >0.7 ng/mL) and Gleason score (4–6 vs. 7–10) were significantly associated with BFFS and DMFS (Table 2). MVA in the EBRT cohort demonstrated that nPSA12 (≤1.0 vs. >1.0 ng/mL), pretreatment PSA (≤10 vs. >10 ng/mL), Gleason score (4–6 vs. 7–10), and T‐stage (T1c–T2a vs. T2b–T3) were significantly associated with BFFS, and nPSA12 and T‐stage were significantly associated with DMFS (Table 3).

**Table 2:** Univariate and multivariate analysis of biochemical failure and distant metastasis in the LDR‐PB cohort

Variables	Biochemical failure			Distant metastasis		
	Univariate	Multivariate		Univariate	Multivariate	
	*P*	*P*	HR	*P*	*P*	HR
Pretreatment PSA (≤10 vs >10 ng/mL)	0.171	0.351	1.589	**0.029**	0.283	1.969
Gleason score (4–6 vs. 7–10)	**0.001**	**0.001**	**5.526**	**0.015**	**0.028**	**4.502**
T‐stage (T1c–T2a vs. T2b–T3)	0.889			0.573		
Total BED (≤210 vs. >210 Gy_2_)	0.303			**0.038**	0.133	3.523
nPSA12 (≤0.7 vs. >0.7 ng/mL)	**0.004**	**0.004**	**6.191**	**0.010**	**0.020**	**11.508**

LDR‐PB, low‐dose‐rate prostate brachytherapy; PSA, prostate‐specific antigen; BED, biologically effective dose; nPSA12, prostate‐specific antigen nadir within 12 months; HR, hazard ratio. Boldface *P* values are statistically significant (< 0.05).

**Table 3:** Univariate and multivariate analysis of biochemical failure and distant metastasis in the EBRT cohort

Variables	Biochemical failure			Distant metastasis		
	Univariate	Multivariate		Univariate	Multivariate	
	*P*	*P*	HR	*P*	*P*	HR
Pretreatment PSA (≤10 vs. >10 ng/mL)	**<0.001**	**0.018**	**2.698**	0.321		
Gleason score (4–6 vs. 7–10)	0.068	**0.030**	**2.972**	0.241	0.195	3.964
T‐stage (T1c–T2a vs. T2b–T3)	**0.001**	**0.010**	**2.641**	**0.001**	**0.013**	**13.930**
Prescribed dose (≤76 vs. ≥78 Gy)	**0.015**	0.060	0.413	0.266		
nPSA12 (≤1.0 vs. >1.0 ng/mL)	**0.001**	**0.005**	**3.516**	**0.023**	**0.041**	**8.741**

EBRT, external beam radiotherapy; PSA, prostate‐specific antigen; nPSA12, prostate‐specific antigen nadir within 12 months; HR, hazard ratio. Boldface *P* values are statistically significant (< 0.05).

In addition, there was a little correlation between pretreatment PSA and nPSA12 in the LDR‐PB cohort (*r* = 0.108, *P* = 0.018) and the EBRT cohort (*r* = 0.260, *P* < 0.001).

## Discussion

The utility of time‐limited nPSA in determining prognosis in patients treated with radiotherapy, especially with LDR‐PB, has not been well examined. We found that lower nPSA12 is related to higher BFFS and DMFS of patients with localized prostate cancer who received LDR‐PB or definitive EBRT without ADT.

Several previous reports have demonstrated that the absolute nPSA after LDR‐PB or EBRT for patients with prostate cancer predicts long‐term clinical outcomes. Ko et al. reported on 921 patients treated with permanent brachytherapy ± EBRT without ADT 15. Five‐year BFFS was 95.2% for patients who achieved nPSA <0.5 ng/mL versus 71.5% for those who did not. In a multi‐institutional study of 4833 patients treated with definitive EBRT without ADT, Ray et al. reported that nPSA and time to nPSA after treatment were predictive of 8‐year BFFS and DMFS in all clinical risk categories 2. However, the clinical utility of nPSA is limited because many patients do not achieve an absolute nPSA for many years after treatment. Moreover, the absolute nPSA is only known after many years of follow‐up. Therefore, we chose nPSA12 as a variable in this study, because we were interested in predicting treatment outcomes at an early, well‐defined time point.

Few retrospective studies have investigated nPSA at early time points after definitive EBRT without ADT as a predictor of treatment outcomes. Zelefsky et al. reported that nPSA ≤1.5 ng/mL at 2 years after EBRT for 844 patients with prostate cancer was an independent predictor of long‐term DM and cause‐specific mortality (CSM), and those achieving nPSA ≤1.5 ng/mL at 2 years were approximately 10% less likely to experience DM compared to those who did not 11. Alcantare et al. reported that in patients who underwent EBRT, nPSA12 ≤ 2.0 ng/mL was an independent predictor of long‐term DM and CSM, but was not significant for BF 10. Ray et al. reported that patients with nPSA12 ≤ 2.0 ng/mL had significantly higher 8‐year BFFS and DMFS (55% and 95%, respectively) than patients with nPSA12 > 2.0 ng/mL (40% and 88%, respectively), and nPSA12 was an independent predictor of BFFS and DMFS 9. In this study, nPSA12 of 1.0 ng/mL was chosen to stratify patients in the EBRT cohort because it was the median nPSA12 after EBRT and there are no guidelines about optimal threshold levels of nPSA at early time points. Our data demonstrate that nPSA12 ≤ 1.0 ng/mL was an independent predictor of long‐term BF and DM, and 7‐year BFFS was 86.1% for patients who underwent EBRT achieving nPSA12 ≤ 1.0 ng/mL, which was 20% higher than the rate of those who did not.

By contrast, there is little evidence about the relationship between time‐limited nPSA and treatment outcomes after LDR‐PB. A few reports have demonstrated that PSA at a fixed time after LDR‐PB for prostate cancer is a useful predictor of treatment outcomes. Lo et al. reported that 4‐year PSA after LDR‐PB for prostate cancer was predictive of long‐term disease‐free survival 16. Stock et al. reported that 5‐year PSA was prognostic, and patients with 5‐year PSA <0.2 ng/mL were unlikely to develop BF 17. However, PSA at 4–5 years after LDR‐PB without ADT is of little use in predicting cancer recurrence, as we found that BF occurred in half of these patients within 4 years. We thought that nPSA12 may be a useful marker for patients treated with LDR‐PB, as has been seen for patients treated with EBRT. In this study, for patients with LDR‐PB achieving nPSA12 ≤ 0.7 ng/mL, the risk of subsequent failure was <1%, meaning that these patients may be reassured of an extremely low chance of relapse. In addition, those achieving nPSA12 ≤ 0.7 ng/mL were approximately 10% more likely to achieve 7‐year biochemical control than those who did not.

There are various treatment options for relapsed prostate cancer after initial radiotherapy according to the location of recurrence. While palliative ADT has been generally used for relapsed prostate cancer, several studies have demonstrated that local recurrence, diagnosed by magnetic resonance imaging and posttreatment biopsy, can be curatively treated by salvage treatment modalities such as prostatectomy, cryosurgery, and low‐ or high‐dose‐rate brachytherapy 18, 19, 20, 21, 22, 23, 24. Some studies have shown that these interventions are more successful when treatment is initiated when PSA levels are lower, reflective of a reduced tumor volume 25, 26. To identify high‐risk patients at an early point using nPSA12 may help relapsed patients have broader salvage treatment options and a chance for cure.

Recently, several markers for prostate cancer other than PSA have been identified. One of these is prostate‐specific membrane antigen (PSMA), a transmembrane glycoprotein that is overexpressed in prostate cancer. Some studies have shown that increased PSMA expression is associated with higher tumor stage and grade and a high risk of disease progression 27, 28. In addition to PSA, it is expected that more accurate prognostic prediction will be possible by combining new biomarkers, such as PSMA.

This study has some limitations. First, it was a retrospective study over a long period (2006–2014). In particular, EBRT techniques and doses changed over this time period. Second, our median follow‐up may be insufficient to assess cancer‐specific and overall survival, and additional failures might be identified with longer follow‐up. Third, it is difficult to directly compare the result between the LDR‐PB and EBRT cohorts because of an imbalance in background prognostic factors between the two cohorts. The EBRT cohort had a higher pretreatment PSA, Gleason score, and T‐stage, which would predict a less favorable outcome. Despite these limitations, our results from this relatively large sample demonstrated that nPSA12 in patients treated with LDR‐PB or EBRT can serve as an early and simple tool that can be used to predict disease progression. Prospective studies with longer follow‐up will fully validate the utility of nPSA12 for predicting the clinical outcomes.

## Conclusion

In conclusion, nPSA12 is an early predictor of disease progression that is independent of pretreatment PSA, radiation dose, and other clinical factors after LDR‐PB or definitive EBRT for patients with localized prostate cancer. Because there are various patterns of clinical failure of prostate cancer after initial radiotherapy, nPSA12 could potentially help identify patients at high risk who might benefit from earlier application of salvage therapy for relapsed prostate cancer.

## Conflict of Interest

The authors made no disclosures.
